# Advances in Immunotherapy and Innovative Therapeutic Approaches for Cancer Treatment: Editorial to the Special Issue “State-of-the-Art Molecular Oncology in Italy”

**DOI:** 10.3390/ijms24108929

**Published:** 2023-05-18

**Authors:** Federica Laudisi, Carmine Stolfi

**Affiliations:** Department of Systems Medicine, University of Rome “Tor Vergata”, 00133 Rome, Italy; federica.laudisi@uniroma2.it

Cancer remains one of the most common causes of death worldwide, mainly due to late diagnosis and the lack of efficient therapeutic options for patients with advanced diseases [1]. Interactions between the immune system and tumors are governed by a complex network of biological pathways [2], and the modulation of immune checkpoints to increase the host immune response against cancer cells represents a recent and valuable therapeutic approach [3]. However, some limits still remain, since only a group of patients respond to immunotherapy, and since some may also develop resistance, immune-mediated adverse effects, and hyper-progressive disease over time [3]. Hence, new therapeutic targets/strategies and personalized treatments are highly desired to improve patient clinical outcomes.

This Special Issue comprises six review articles and six original studies on advances in cancer immunotherapy, as well as the characterization of new molecular targets and the genomic and transcriptomic profiles of cancer patients to develop efficient and personalized therapeutic strategies.

Programmed cell death protein 1 (PD-1) is a transmembrane protein that is expressed mainly by cells of the immune system, such as T lymphocytes, and plays a crucial role in immune self-tolerance [4]. Increased expression of the programmed cell death protein ligand 1 (PD-L1) by cancer cells and the consequent aberrant activation of the PD-1/PD-L1 axis and inhibition of T cell antitumor activity constitute one of the main mechanisms of immune evasion [4]. In this context, the use of the PD-1 inhibitors pembrolizumab and nivolumab in head and neck squamous cell carcinoma (HNSCC) represents a valuable therapeutic strategy with a better overall survival (OS) and a better safety profile, although most patients obtain beneficial effects only for a short duration [5]. In this sense, the use of different biomarkers may help in predicting successful responses to anti-PD-1/PD-L1 treatment. Meliante et al. highlighted the importance of interferon (IFN)-ɣ cytokine signaling, the head and neck cancer-associated fibroblast profile, the presence of a high tumor mutation burden and hypoxia-inducible factor-1 (HIF-1)-mediated signaling, which correlate with the hypoxia level in tumor microenvironments, as important biomarkers that can predict the clinical response to the PD-1 blockade in HNSCC [6]. In addition, drug combinations with new molecules may be used to overcome the lack of response to the anti-PD-1/PD-L1 treatment. For example, monalizumab is a humanized monoclonal antibody capable of unleashing the antitumor immune response by targeting NKG2A, a novel checkpoint inhibitor expressed on cytotoxic lymphocytes, including natural killer (NK) cells and subsets of activated CD8+ T cells. GSK609, in contrast, is a humanized antibody that is capable of boosting T-cell proliferation and survival thanks to its potent agonist activity against the inducible T-cell co-stimulator (ICOS). Both antibodies are currently being tested in phase III clinical trials for the treatment of HNSCC [6].

The use of immune checkpoint inhibitors has also increased and improved the treatment options for patients with non-oncogene-addicted advanced stage non-small-cell lung cancer (NSCLC), whereas their therapeutic role in oncogene-addicted advanced stage NSCLC is still debated as many patients fail to respond. In a retrospective study carried out at the University of Naples Federico II, Pisapia and colleagues retrospectively evaluated data collected from their archives of advanced stage NSCLC patients with positive PD-L1 expression (1%) (*n* = 167) for at least five of the most common driver mutations, namely, Epidermal Growth Factor Receptor (*EGFR*), V-Raf Murine Sarcoma Viral Oncogene Homolog B1 (*BRAF*), Kirsten Rat Sarcoma Viral Oncogene Homolog (*KRAS*), Anaplastic Lymphoma Receptor Tyrosine Kinase (*ALK*) and ROS Proto-Oncogene 1, Receptor Tyrosine Kinase (*ROS1*), to provide a molecular landscape of clinically relevant oncogenic drivers in PD-L1-positive NSCLC patients in these subjects [7]. More than half of the patients (*n* = 93) presented at least one genomic alteration and the results clearly showed a positive correlation between PD-L1 expression and *KRAS* mutations in 56 patients, with some of them displaying increased sensitivity to treatment with pembrolizumab [7]. 

Anti-PD-1 therapies are particularly efficient in patients with gastric adenocarcinomas characterized by specific molecular profiles, such as microsatellite instability-high (MSI-h)/deficient mismatch repair (dMMR) profiles, Epstein–Barr virus (EBV)^+^, and a high level of PD-L1 [8,9,10]. Unfortunately, a considerable proportion of biomarker-negative patients must resort to standard therapies. In their retrospective multicenter study, Salati et al. investigated the effect of first-line chemotherapy (plus or minus the anti-human epidermal growth factor receptor 2 (HER2) agent trastuzumab, according to the HER2 status) and anti-PD-1 treatment against gastric adenocarcinoma and gastro-esophageal junction adenocarcinoma in a newly diagnosed Western cohort characterized by subtype-defining biomarkers (i.e., d-MMR, EBV^+^, HER2^+^, and all-negative biomarkers) [11]. The results showed that some subgroups of patients, such as those with dMMR and EBV^+^, and those with a high PD-L1 status (according to the combined positive score of ≥5), showed longer overall survival compared to the other groups [11].

Beyond characterizing the molecular profile of cancer patients, the efficacy of immunotherapy can also be improved once combined with selected anthelmintic drugs [12], which have already shown important antineoplastic effects [13]. In this regard, the halogenated salicylanilide compound niclosamide was found to affect Signal Transducer and Activator of Transcription 3 (STAT3) phosphorylation and related PD-L1 expression in oncogene-addicted advanced-stage NSCLC patients, while flubendazole, which belongs to the benzimidazole family, exerted similar effects on STAT3, affecting PD-1 expression. Another benzimidazole compound, albendazole, was able to target ubiquilin-4, a protein that interacts with and stabilizes PD-L1, thus promoting its proteasomal degradation [12]. Some anthelmintic agents (rafoxanide and ivermectin) also recently revealed their ability to cause some tumors types to be “immunogenic” via the promotion of a particular form of apoptosis called immunogenic cell death, which allows cancer cells to be recognized and targeted by the immune system [12].

In addition to the need to increase responsiveness to immunotherapy, another significant goal for cancer treatment at the early and late stages is the characterization of new therapeutic molecular targets on tumor cells that can improve treatment efficacy and limit the potential side effects on non-tumor cells. In this regard, Busato and coworkers provided a comprehensive overview of antibody-based immunotherapy and antibody-conjugated nanoparticle-based targeted therapy to affect the tumor-associated antigen glypican-1 (GPC1) in patients with pancreatic ductal adenocarcinoma (PDAC) [14]. This proteoglycan is highly expressed in tumor cells, while it is absent in healthy tissue and chronic pancreatitis, thus offering a personalized therapeutic approach against malignant cells [14].

Another member of the glypican family, glypican-3 (GPC3), is now emerging as an important tumor-associated antigen that can be targeted for the treatment of cancer patients [15]. Mossenta et al. proposed that GPC3 is a valuable target for the treatment of advanced hepatocellular carcinoma (HCC) [15]. In particular, GPC3 is a heparan sulfate proteoglycan that sustains hepatocarcinogenesis through the modulation of glucose metabolism, via the upregulation HIF-1 expression and lactate production, and several growth factors [16]. The authors considered GPC3 a promising molecular target for the diagnosis of HCC and a valuable candidate for nanoparticle-based therapies and drug delivery approaches owing to its specificity to HCC tissue, thus overcoming drug resistance [15].

Ferraro et al. have characterized a novel potential therapeutic target for the treatment of glioblastoma multiforme (GBM) [17]. In their study, the authors identified brain glycogen phosphorylase (PYGB) as a valid molecular target for the treatment of GBM, which catalyzes the transformation from glycogen to glucose 1-phosphate and supports glucose metabolism in cancer cells (the so-called “Warburg effect”). The results of a combined biochemical and proteomic analysis revealed that PYGB could be targeted in different GBM cell lines and primary neuronal cells via 2,3-benzodiazepin-4-one, a blood–brain barrier-permeating compound, also known as 1g, that interferes with cancer cell metabolism [17].

In the context of GBM, the peritumoral brain zone (PBZ) is now considered an influential player in tumor maintenance and progression, as well as in relapse occurrence [18]. The aim of the study by Giambra et al. was to better characterize this apparently unaffected area to identify differentially expressed genes compared to those in tumor tissue [19]. The results of the genomic profiles of the matched tumor core and PBZ biopsies indicated that the cyclin-dependent kinase-4 (CDK4) and exostosin glycosyltransferase-2 (*EXT2*) genes were important players in the early stages of the malignant transformation of PBZ and reported a negative correlation between the increased expression of CDK4 in PBZ and reduced overall survival [19]. Although they are only preliminary, these data constitute a good premise for future investigations into the possible role of CDK4 and *EXT2* in the malignant transformation of PBZ.

Along with the molecules mentioned above, significant advances have been made in the characterization and development of antitumor drugs against mitochondria [20]. In fact, several experimental pieces of evidence support the notion of mitochondrial involvement in the chemoresistance and radioresistance of cancer cells, as it can induce the release of reactive oxygen species and consequent DNA mutations, the transformation of mitochondrial outer membrane permeability, and an abnormal accumulation of mitochondrial metabolites [21,22,23]. In addition to these mechanisms, mitochondrial epigenetics represent another critical aspect that still needs to be fully explored to develop efficient antitumor therapeutic strategies. In this regard, Zaffaroni et al. provided a complete overview of the possible implications of mitochondria in cancer radioresistance and meaningful updates on emerging mitochondria-targeting agents, which confer beneficial antitumor effects once combined with standard therapies [24].

As discussed previously, the discovery of new molecular targets for tumor treatment also relies on a detailed characterization of the genomic and transcriptional profiles of each cancer patient to develop a “personalized” therapy. In their pilot study, Lastraioli et al. focused their attention on *BRAF* wild-type metastatic melanoma, which currently lacks an efficient targeted therapy [25]. The authors compared the gene expression of six independent tumors (all progressed towards metastatic disease) to the expression profile of non-dysplastic melanocytes (considered a healthy control) using gene set enrichment analysis (GSEA) and RNA-Seq data retrieved from the TCGA/GTEx databases. The results showed a distinct upregulation of the genes involved in the immune response (i.e., major histocompatibility complex (MHC) class I and class II pathways) and proteasome activity, as well as transcripts related to mitoribosome proteins, whereas those related to cytosolic ribosome proteins showed decreased expression compared to that of healthy controls [25]. Although this pilot study analyzed a small group of patients, the immune profiles obtained by the authors may potentially help in predicting the responsiveness of *BRAF* wild-type metastatic melanoma to immunotherapy and highlight the importance of protein synthesis and degradation for cancer cell survival in this pathology, thus allowing the identification of the best therapeutic approach for these patients [25].

Detecting gene alterations in cell-free circulating DNA/RNA via liquid biopsy may also be considered a helpful approach to integrate with conventional diagnostic and therapeutic programs. In their study, Lupini et al. investigated the importance of liquid biopsy in the clinical management of patients with NSCLC by using next-generation sequencing (NGS) and assessed whether or not this method could also be applied at earlier stages of the disease, including the first diagnosis [26]. The results obtained from 641 plasma samples of 57 patients allowed the detection of mutations in circulating DNA/RNA 80 days prior to disease progression, including 13 de novo mutations [26]. However, the low amount of circulating DNA/RNA at the early stage of the disease severely limits the usefulness of this method of analysis for these subjects, although the sensitivity of this method is improving over time. Although liquid biopsy cannot replace tissue biopsy, it represents a useful tool once combined with conventional diagnostic investigations in diagnosis and imaging approaches used during disease progression.

As aforementioned, cancer remains one of the most common causes of death not only due to the shortage of efficient therapeutic approaches, but also due to late diagnosis, as is the case with certain types of malignancies. Pancreatic ductal adenocarcinoma is a prime example, as it is mostly asymptomatic in the early stages and is unfortunately diagnosed too late for curative treatment. In their review, Caputo and colleagues provide a complete overview of their most significant discoveries in the field of PDAC detection and propose the use of the protein corona, i.e., nanomaterials coated with plasma proteins from biological fluids, as a promising tool for early cancer detection [27]. They developed nano-enabled blood (NEB) tests that can discriminate between the protein corona of PDAC patients, those of healthy subjects and subjects with other cancer types, as they have different charges and compositions, or rather a unique signature associated with the type and stage of the tumor [27]. However, even if NEB tests may represent a promising early diagnostic tool for PDAC patients and may be included in common clinical practice, additional efforts must be devoted to improving data reproducibility and standardizing the experimental protocols.

Personalized therapy remains one of the most important and concern-provoking challenges in overcoming all of the limitations faced in clinical practice with regard to the management of cancer patients, such as late diagnosis, acquired resistance, and cytotoxic side effects. The original articles and reviews proposed in this Special Issue aim to describe and discuss the recent advances in immunotherapy and innovative therapeutic targets in cancer treatment in Italy. The discovery of new molecular targets with which to predict and improve patient response to immunotherapy, the characterization of the genomic and transcriptomic profiles of patients, the identification of innovative therapeutic targets in cancer treatment beyond immunotherapy, as well as the development of alternative nanotechnologies to ensure early-stage cancer diagnosis are all crucial aspects to be considered in order to improve clinical outcomes. However, there is still much work to be conducted, and further studies are still required to confirm and validate such innovative therapeutic approaches in common clinical practice.

## Data Availability

Not applicable.
